# Interrelationship of Postoperative Delirium and Cognitive Impairment and Their Impact on the Functional Status in Older Patients Undergoing Orthopaedic Surgery: A Prospective Cohort Study

**DOI:** 10.1371/journal.pone.0110339

**Published:** 2014-11-17

**Authors:** Chih-Kuang Liang, Chin-Liang Chu, Ming-Yueh Chou, Yu-Te Lin, Ti Lu, Chien-Jen Hsu, Liang-Kung Chen

**Affiliations:** 1 Center for Geriatrics and Gerontology, Kaohsiung Veterans General Hospital, Kaohsiung, Taiwan; 2 Division of Neurology, Department of Medicine, Kaohsiung Veterans General Hospital Kaohsiung, Taiwan; 3 Aging and Health Research Center, National Yang Ming University, Taipei, Taiwan; 4 Department of Psychiatry, Kaohsiung Veterans General Hospital, Kaohsiung, Taiwan; 5 Department of Family Medicine, Kaohsiung Veterans General Hospital, Kaohsiung, Taiwan; 6 Department of Orthopaedics, Kaohsiung Veterans General Hospital, Kaohsiung, Taiwan; 7 Center for Geriatrics and Gerontology, Taipei Veterans General Hospital, Taipei, Taiwan; Nathan Kline Institute and New York University School of Medicine, United States of America

## Abstract

**Background:**

The impact of postoperative delirium on post-discharge functional status of older patients remains unclear, and little is known regarding the interrelationship between cognitive impairment and post-operative delirium. Therefore, the main purpose was to evaluate the post-discharge functional status of patients who experience delirium after undergoing orthopaedic surgery and the interrelationship of postoperative delirium with underlying cognitive impairment.

**Method:**

This prospective cohort study, conducted at a tertiary care medical center from April 2011 to March 2012, enrolled all subjects aged over 60 years who were admitted for orthopaedic surgery. The baseline characteristics (age, gender, BMI, and living arrangement), surgery-related factors (ASA class, admission type, type of surgery, and length of hospital stay), results of geriatric assessment (postoperative delirium, cognition, depressive mood, comorbidity, pain, malnutrition, polypharmacy, ADL, and instrumental [I]ADL) and 1–12-month postoperative ADL and IADL functional status were collected for analysis.

**Results:**

Overall, 9.1% of 232 patients (mean age: 74.7±7.8 years) experienced postoperative delirium, which was significantly associated with IADL decline at only 6 and 12 months postoperatively (RR: 6.22, 95% CI: 1.08–35.70 and RR: 12.54, 95% CI: 1.88–83.71, respectively). Delirium superimposed on cognitive impairment was a significant predictor for poor functional status at 6 and 12 months postoperatively (RR: 12.80, 95% CI: 1.65–99.40 for ADL at the 6^th^ month, and RR: 7.96, 95% CI: 1.35–46.99 at the 12^th^ month; RR: 13.68, 95% CI: 1.94–96.55 for IADL at the 6^th^ month, and RR: 30.61, 95% CI: 2.94–318.54 at the 12^th^ month, respectively).

**Conclusion:**

Postoperative delirium is predictive of IADL decline in older patients undergoing orthopaedic surgery, and delirium superimposed on cognitive impairment is an independent risk factor for deterioration of ADL and IADL functional status. Early identification of cognitive function and to prevent delirium are needed to improve functional status following orthopaedic surgery.

## Introduction

Delirium is an acute mental disorder that is characterized by rapid onset and a fluctuating course of consciousness disturbance and inattention, possibly leading to further adverse health consequences in the elderly population [1]. Although delirium is a well-recognized geriatric syndrome, it is often overlooked by clinicians and nurses [2], [3]. The incidence of delirium following orthopaedic surgery has been reported to be 4–65%. Considerable variation is seen and is dependent on the type of procedure, with the reported incidence being 35–65% in patients undergoing operative treatment of a hip fracture and 9–15% in patients undergoing elective orthopaedic procedures [4], [5]. Risk factors for the development of postoperative delirium in older patients include older age, cognitive impairment, depressed mood, poor baseline physical function, comorbid diseases, type of surgery, and institutionalization before admission [6]–[10]. Although delirium is a common geriatric syndrome, its etiopathogenesis remains unclear. However, preventive strategies focused on early identification and management of risk factors are believed to be superior to strategies that emphasize treatment of delirium after it occurs [10]–[13].

Delirium subsequent to surgery is associated with higher rates of in-hospital and long-term mortality [14]–[20], as well as longer hospital stay, longer intensive care unit stay, and higher chance of discharge to nursing facilities [14]–[16], [21]. Although the adverse impact of postoperative delirium has been clearly identified, the impact of postoperative delirium on long-term functional status remains unclear. Dementia or cognitive impairment has been reported as an independent risk factor for delirium [10], and the overall incidence of new delirium was significantly higher among older patients with dementia than among older patients with no dementia [22]. Moreover, pre-fracture cognitive impairment and post-fracture delirium were also strongly associated with higher mortality rate and risk for institutionalization [23], [24], and delirium might be an early indicator for post-discharge cognitive decline [25], [26]. Although it can result in long-term cognitive decline, postoperative cognitive decline secondary to delirium does not occur in all patients [10]. To date, little is known regarding the interrelationship between delirium and cognitive impairment and their impact on adverse functional status in older patients. Therefore, the purpose of the present study was to evaluate the impact of postoperative delirium, in the presence of underlying cognitive impairment, on changes in the post-discharge functional status of patients who underwent orthopaedic surgery.

## Methods

### Study design

This prospective cohort study was conducted in a tertiary care medical center in Southern Taiwan. All subjects aged 60 years and older who were admitted for orthopaedic surgery during the period April 2011 to March 2012 were screened for this study. Patients were excluded for the following reasons: (1) medical conditions that prevented comprehensive geriatric assessment (CGA), or admission or transfer to an intensive care unit before enrollment, (2) inability to complete the comprehensive geriatric assessment (CGA), (3) inability to provide informed consent, (4) limited life expectancy less than 6 months such as in cancer and terminal stage heart failure cases, (5) delirium occurring before enrollment or surgery, and (6) incomplete data. The study protocol was approved by the Institutional Review Board of Kaohsiung Veterans General Hospital and written informed consents were obtained from all participants before the study started. During a one-year period, a total of 232 patients were enrolled with mean age of 74.7±7.8 years (range: 60–93). Among them, 28 (12.1%) patients were admitted to the hospital from the emergency department. The incidence of postoperative delirium was 9.1% (21/232).

### Preoperative evaluation

#### Demographic data and surgery characteristics

Two research nurses interviewed all participants to collect the pre-operative demographic data (including age, gender, educational level, living arrangement, and body mass index [BMI]), and data on other characteristics including admission type (emergency or elective surgery), type of surgery (including spinal decompression only, spinal surgery with instrumented fusion, total knee arthroplasty, other elective knee surgery, elective total hip arthroplasty [total hip replacement]/bipolar hemiarthroplasty, revision hip surgery, and open reduction and internal fixation [ORIF]/arthroplasty for hip fracture), ASA (American Society of Anesthesiologists) physical status, and length of hospital stay.

#### Comprehensive geriatric assessment (CGA)

All participants were assessed by trained research nurses using the CGA before preceding the orthopaedic operation, and within the first 24 hours after hospital admission from the outpatient clinics or emergency department. The assessment covered visual and hearing impairments, polypharmacy (defined as currently using >4 prescription drugs for over 2 weeks), depressive symptoms (using the 15-item Chinese Geriatric Depression Scale) [27], [28], nutritional status (as determined by the Mini Nutritional Assessment) [29], comorbidity (as evaluated by the Charlson's Comorbidity Index) [30], symptoms of pain (rated on a Visual Analogue Scale) [31], cognitive function (as assessed by the Chinese version of the Mini-Mental State Examination at admission) [32], the Activities of Daily Living (ADL, evaluated by the Barthel Index) [33], and the Lawton-Brody Instrumental ADL (IADL) [34].

Patients were screened for delirium daily by the primary care nurses in the orthopaedic wards after orthopaedic surgery using the Confusion Assessment Method (CAM) [35], and a senior psychiatrist would confirm the diagnosis if the patient was deemed to be confused. The research nurses obtained ADL and IADL scores by conducting telephone interviews of the participating patients or their primary caregivers at 1, 3, 6, and 12 months after hospital discharge.

### Statistical analysis

Continuous data are expressed as means ± SD, and categorical data are expressed as percentages. Dichotomous and ordinal variables were compared in those with and without delirium using the Chi-square test or Fisher exact test, and continuous variables were compared using the independent Student *t*-test or Mann-Whitney *U* test when appropriate.

The independent Student *t*-test or Mann-Whitney *U* test were used to compare the ADL or IADL scores between groups based on delirium status at 1, 3, 6, and 12 months. The differences in mean ADL and IADL scores between those with and without delirium at different time points were evaluated using the Analysis of Covariance (ANCOVA) with the inclusion of baseline ADL or IADL scores as covariates. To compare functional changes at 1, 3, 6, and 12 months, ADL or IADL functional decline was defined as lower ADL or IADL score at follow-up than at baseline. Multivariable logistic regression analysis was performed to determine whether delirium was an independent predictor of ADL or IADL decline at follow-up. In this model, age, gender, admission type, type of surgery, hearing impairment, polypharmacy, cognitive impairment, depressive symptoms, BMI, high risk of malnutrition, Charlson's Comorbidity Index (CCI), symptoms of pain, ASA class, and hospital length of stay were entered as explanatory variables.

To evaluate the effect of cognitive problems on long-term functional status (ADL and IADL scores at 1, 3, 6, and 12 months follow-up), four categorical variables representing four groups were defined (no cognitive problem [group A]; cognitive impairment alone indicated by MMSE <24 [group B]; postoperative delirium superimposed on cognitive problems [group C], and delirium only [group D]). ANCOVA was used to compare the mean ADL and IADL scores between these four groups after adjusting for baseline ADL and IADL scores (the covariates). The independent effect of each categorical variable on ADL and IADL outcomes was assessed using multivariable logistic regression analysis after controlling for age, gender, admission type, type of surgery, hearing impairment, polypharmacy, cognitive impairment, depressive symptoms, BMI, high risk of malnutrition, CCI, symptoms of pain, ASA class, and hospital length of stay and baseline IADL or ADL scores as covariates. For all tests, a P value (two-tailed) less than 0.05 was considered statistically significant. All statistical analyses were performed using IBM SPSS version 21 (SPSS Inc., Chicago, IL).

## Results

### The effect of delirium on functional status

Demographic characteristics of all 232 patients are summarized in Table 1. Fifty of the 232 patients were lost to follow up and therefore excluded from the evaluation of post-discharge functional status. The demographic characteristics of the excluded patients were similar to those of the enrolled patients except for age (77.0±8.7 years vs 74.1±7.4 years, p = 0.018) and BMI (25.2±4.5 vs 26.7±4.1 kg/m^2^, p = 0.015). The number of patients who underwent spinal decompression only, spinal surgery with instrumented fusion, total knee arthroplasty, other elective knee surgery, elective total hip replacement/bipolar hemiarthroplasty, revision hip surgery, and ORIF/arthroplasty for hip fracture was 7 (3.0%), 53 (22.8%), 92 (39.7%), 8 (3.4%), 31 (13.4%), 8 (3.4%), and 33 (14.2%), respectively. These procedures were grouped into four categories (elective spine surgery, elective knee surgery, elective hip arthroplasty, and ORIF/arthroplasty for hip fracture) for further analysis. Comparing the 50 patients lost to follow up with the 182 included patients, the delirium occurred in a similar proportion of both groups (8.0% [4/50] vs 9.3% [17/182]; data not shown), indicating that delirium did not increase the likelihood of loss to follow up.

**Table 1:** Demographic data, functional status, and surgery-related factors in 232 subjects with postoperative delirium or no delirium.

	Total	Postoperative delirium	No delirium	
	N = 232	N = 21	N = 211	
Variables	(% or mean ± SD)	(% or mean ± SD)	(% or mean ± SD)	*p* value
Age	74.7±7.8	81.3±5.2	74.1±7.7	<0.001
Educational level	5.8±4.7	6.4±4.6	5.7±4.7	0.522
Gender (male)	108(46.6%)	17(81.0%)	91(43.1%)	0.001
Admission route				0.149
Emergency room	28(12.1%)	5(23.8%)	23(10.9%)	
Outpatient clinic (elective)	204(87.9%)	16(76.2%)	188(89.1%)	
Living				0.035
Alone	32(13.8%)	1(4.8%)	31(14.7%)	
Institutionalized	10(4.3%)	3(14.3%)	7(3.3%)	
With relatives/friends	190(81.9%)	17(81.0%)	173(82.0%)	
BMI	26.5±4.3	23.7±3.2	26.8±4.2	0.001
Polypharmacy (Yes)	106(45.7%)	13(61.9%)	93(44.1%)	0.118
Psychotic drugs (Yes)	36(15.5%)	4(19.0%)	32(15.2%)	0.751
Visual impairment (Yes)	157(67.7%)	18(85.7%)	139(65.9%)	0.064
Hearing impairment (Yes)	38(16.4%)	8(38.1%)	30(14.2%)	0.010
ADL(BI) before admission	94.1±2.3	85.0±19.9	95.0±1.0	0.034
IADL before admission	5.7±1.4	4.4±1.9	5.8±1.3	0.004
Cognitive impairment at admission (MMSE <24)	91(39.2%)	16(76.2%)	75(35.5%)	<0.001
Presence of depressive symptoms (defined by GDS-15> = 5)	19(8.2%)	1(4.8%)	18(8.5%)	1.000
Risk of malnutrition (screening by MNA)	19(8.2%)	7(33.3%)	12(5.7%)	<0.001
CCI	0.82±1.07	1.71±1.79	0.73±0.93	0.022
Pain VAS score	4.72±1.58	5.14±1.71	4.68±1.56	0.203
ASA				0.124
ASA 1 and 2	225(97.0%)	19(90.5%)	206(97.6%)	
ASA 3	7(3.0%)	2(9.5%)	5(2.4%)	
Length of hospital stay	8.45±4.46	8.42±4.63	8.76±2.221	0.736
Type of surgery				0.090
Elective spine surgery	60(25.9%)	7(11.7%)	53(88.3%)	
Elective knee surgery	100(43.1%)	6(6.0%)	94(94.0%)	
Elective hip arthroplasty	39(17.7%)	1(2.6%)	38(97.4%)	
ORIF/arthroplasty for hip fracture	33(13.4%)	7(21.2%)	26(78.8%)	

BMI: Body Mass Index; ADL: Activities of Daily Living; BI: Barthel Index; IADL: Instrumental Activities of Daily Living; MMSE: Mini-mental State Examination; GDS: Geriatric Depression Scale; MNA: Mini-nutritional Assessment; CCI: Charlson's Comorbidity Index; VAS: Visual Analogue Scale; ASA: American Society of Anesthesiologists physical status; ORIF: open reduction and internal fixation.

Elective Spine Surgery: Spinal decompression only and Spinal surgery with instrumented fusion.

Elective knee surgery: includes total knee replacement (N = 92) and other elective knee surgery (N = 8).

Elective hip arthroplasty: includes total hip replacement, bipolar hemiarthroplasty, and revision hip surgery.

**Figure 1:**
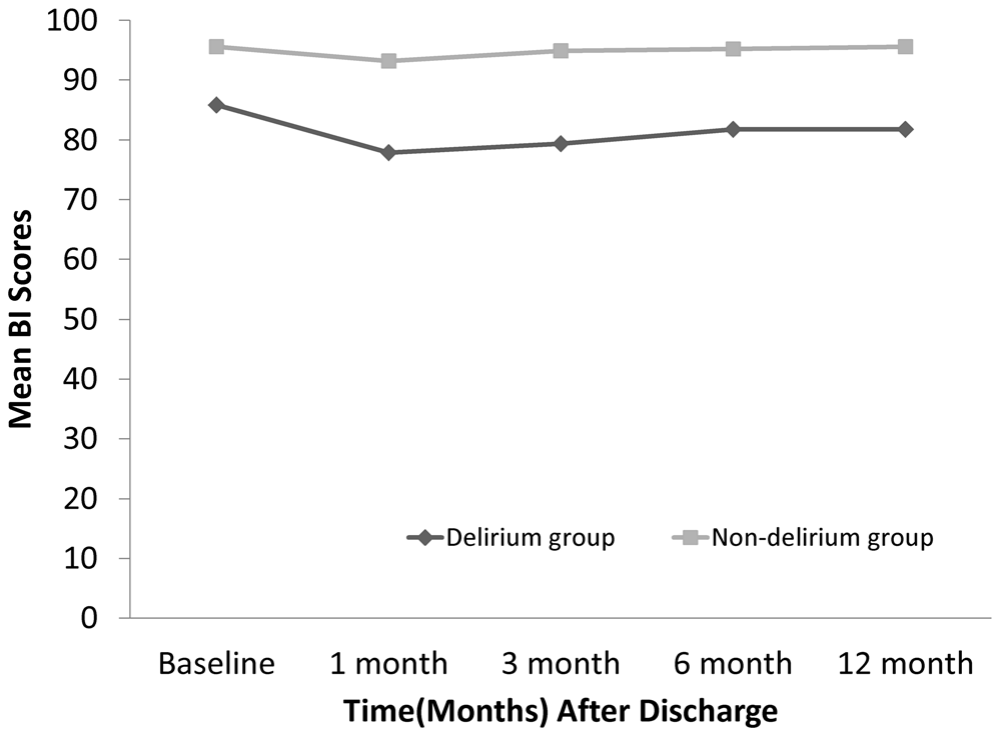
The trend of Barthel Index scores (ADL) (a) and IADL (b) for the delirium and no delirium groups.

At baseline, the 182 enrolled subjects (17 with delirium and 165 without delirium) had an ADL score of 85.9 (standard error [SE] = 4.81) vs 95.6 ([SE] = 0.80) and an IADL score of 4.4 ([SE] = 0.40) vs 5.8 ([SE] = 0.09; Table 2). Figure 1 shows the change in ADL and IADL scores over time after hospitalization for both groups. ADL and IADL scores were significantly different between those who developed post-operative delirium and those who did not. When controlling for baseline ADL, IADL scores remained significantly lower in patients who developed postoperative delirium than in those who did not. Table 3 shows the differences in ADL and IADL decline between the two groups at different follow-up times. Results of univariate logistic regression analysis identified delirium as a predictor of ADL decline at 6 and 12 months (p value: 0.036 vs 0.014), and IADL decline at 3, 6, and 12 months after surgery (p value: 0.033 vs 0.005 vs 0.001). In addition, multivariable logistic regression analysis identified delirium as a predictor of only IADL decline at 6 and 12 months (for IADL at 6 months, adjusted RR: 6.22, 95% CI:1.08–35.70, p value  = 0.040; for IADL at 12 months, adjusted RR: 12.54, 95% CI: 1.88–83.71, p value  = 0.009).

**Table 2:** Comparing the ADL and IADL scores at baseline and 1, 3, 6, and 12 months of follow-up in groups with delirium.

	Time of follow-up (months)								
	Non-adjusted functional status (mean ± SE)					Adjusted baseline functional status by ANCOVA (mean ± SE)			
Outcomes variables	Baseline	1	3	6	12	1	3	6	12
ADL (BI) scores (mean ± SE)									
Delirium (N = 17)	85.9±4.81#	77.9±5.25#	79.4±5.62#	81.8±5.44#	81.8±5.86#	83.0±2.82#	84.5±2.77#	86.9±3.06#	86.4±3.18#
No delirium (N = 165)	95.6±0.80	93.2±0.93	94.9±0.89	95.2±1.00	95.6±0.99	92.7±0.88	94.4±0.87	94.6±0.96	95.2±1.00
IADL scores (mean ± SE)									
Delirium (N = 17)	4.4±0.40#	3.4±0.45#	3.4±0.47#	3.2±0.53#	2.9±0.53#	4.4±0.28#	4.4±0.28#	4.3±0.33#	4.0±0.33#
No delirium (N = 165)	5.8±0.09	5.3±0.11	5.6±0.11	5.6±0.12	5.7±0.12	5.2±0.09	5.5±0.09	5.5±0.10	5.6±0.10

# p<0.05 for comparing groups by delirium status. ANCOVA: Analysis of Covariance.

**Table 3:** Analysis of the percentage of functional decline and the predicted effect on functional status in groups divided by delirium status using Chi-square test and multivariable logistic regression.

	Percentage of functional decline						Predicted effect on functional decline											
	ADL decline			IADL decline			RR of delirium for ADL decline						RR of delirium for IADL decline					
	Delirium	No delirium		Delirium	No delirium		Unadjusted			Adjusted			Unadjusted			Adjusted		
Follow-up times	N(%)	N(%)	p value	N(%)	N(%)	p value	RR	95% CI	p value	RR	95% CI	p value	RR	95% CI	p value	RR	95% CI	p value
1-month	8/17(47. 1)	46/165(27.9)	0.099	6/13(46.2%)	59/165(35.8)	0.552	2.30	0.84–6.32	0.107	2.45	0.60–9.99	0.210	2.02	0.74–5.52	0.170	1.84	0.47–7.12	0.380
3-month	5/17(29.4)	25/165(15.2)	0.164	6/13(46.2%)	45/165(27.3)	0.045	2.33	0.76–7.20	0.141	1.85	0.36–9.68	0.465	3.00	1.09–8.25	0.033	2.75	0.66–11.47	0.164
6-month	6/15(40.0)	27/160(16.9)	0.040	7/13(53.8)	41/165(24.8)	0.045	3.28	1.08–9.99	0.036	3.68	0.64–21.18	0.144	4.32	1.55–12.08	0.005	6.22	1.08–35.70	0.040
12-month	7/17(41.2)	26/165(15.8)	0.017	8/13(61.5)	39/165(23.6)	0.006	3.74	1.31–10.72	0.014	3.55	0.70–17.91	0.125	5.92	2.06–17.06	0.001	12.54	1.88–83.71	0.009

Covariates after adjusting for ADL: age, gender, admission type, type of surgery, hearing impairment, polypharmacy, MMSE scores, GDS-15 scores, BMI, risk of malnutrition, CCI scores, pain VAS scores, ASA physical status, length of hospital stay, baseline IADL scores.

Covariates after adjusting for IADL: age, gender, admission type, type of surgery, hearing impairment, polypharmacy, MMSE scores, GDS-15 scores, BMI, risk of malnutrition, CCI scores, pain VAS scores, ASA physical status, length of hospital stay, baseline ADL scores.

### Impact of combined cognitive problems on functional status

**Figure 2:**
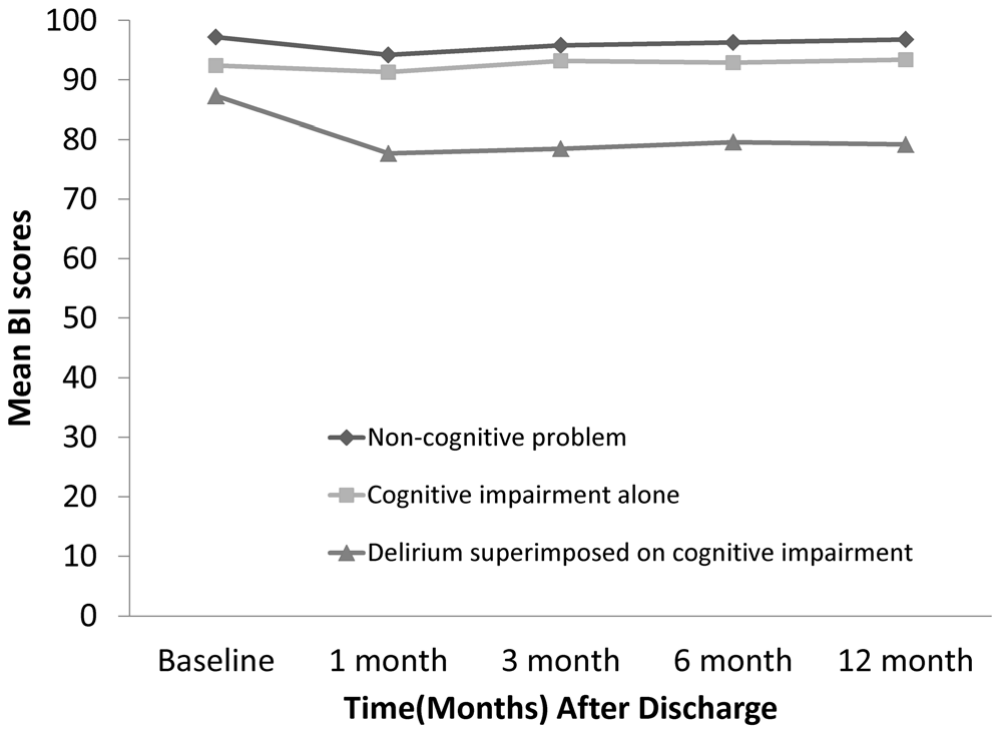
The trend of Barthel Index scores (ADL) (a) and IADL (b) for three groups corresponding to non-cognitive problem, cognitive impairment alone, and delirium superimposed on cognitive impairment.

The 182 included patients were divided into four groups based on delirium and cognitive status. The patients in group D (with delirium, but without baseline cognitive impairment) were excluded because of small sample size (N = 4). Baseline ADL and IADL scores were significantly different between patients with no cognitive problems (group A), and those with both baseline cognitive dysfunction and delirium (group C) or with baseline cognitive dysfunction only (group B; Table 4). In terms of the decline in ADL and IADL scores at 1, 3, 6, and 12 months of follow up, ANCOVA (after adjustment for baseline ADL and IADL values) found significant ADL and IADL decline only in group C (Figure 2). After controlling for confounders (Table 5), multivariable logistic regression still showed the tendency for ADL and IADL to decline in patients in group C at 6 and 12 months (Adjusted RR for ADL at 6 months: 12.80, 95% CI: 1.65–99.40; Adjusted RR for ADL at 12 months: 7.96, 95% CI: 1.35–46.99; Adjusted RR for IADL at 6 months: 13.68, 95% CI: 1.94–96.55; Adjusted RR for IADL at 12 months: 30.61, 95% CI: 2.94–318.54).

**Table 4:** Comparing the ADL and IADL scores at baseline and 1, 3, 6, and 12 months of follow-up in groups divided on the basis of cognitive problems.

	Time of follow-up (months)								
	Non-adjusted functional status (mean ± SE)					Adjusted baseline functional status by ANCOVA (mean ± SE)			
Outcomes variables	Baseline	1	3	6	12	1	3	6	12
ADL (BI) scores (mean ± SE)^ a,b^									
Non-cognitive problems (A), N = 109	97.2±0.71^$,^ *	94.2±1.10*	95.8±1.05*	96.3±1.00*	96.8±0.89*	93.0±1.10*	94.5±1.08*	95.0±1.19*	95.6±1.24*
Cognitive impairment alone (B), N = 56	92.4±1.85^$^	91.3±1.68^#^	93.2±1.62^#^	92.9±2.19^#^	93.4±2.32^#^	92.6±1.53^#^	94.6±1.51^#^	94.4±1.66^#^	94.8±1.72^#^
Delirium superimposed on cognitive impairment (C), N = 13	87.3±5.36*	77.7±6.01* ^,#^	78.5±6.76* ^,#^	79.6±6.83* ^,#^	79.2±7.42* ^,#^	81.8±3.21* ^,#^	82.7±3.16* ^,#^	84.2±3.47* ^,#^	83.3±3.61* ^,#^
IADL scores (mean ± SE)^ a,b^									
Non-cognitive problems (A), N = 109	6.1±0.09* ^,$^	5.7±0.12* ^,$^	5.8±0.13* ^,$^	5.9±0.14* ^,$^	6.0±0.14* ^,$^	5.4±0.11*	5.5±0.11*	5.6±0.13*	5.7±0.13*
Cognitive impairment alone (B), N = 56	5.3±0.20^#,$^	4.7±0.20^#,$^	5.0±0.21^#,$^	5.0±0.22^#,$^	5.1±0.22^#,$^	5.0±0.15^#^	5.4±0.15^#^	5.3±0.18*	5.4±0.18^#^
Delirium superimposed on cognitive impairment (C), N = 13	4.3±0.47* ^,#^	3.3±0.49* ^,#^	3.2±0.51* ^,#^	3.1±0.60* ^,#^	2.7±0.58* ^,#^	4.4±0.32* ^,#^	4.4±0.32* ^,#^	4.2±0.38* ^,#^	3.7±0.38* ^,#^

^*^p<0.05 for comparing C and A; ^#^p<0.05 for comparing C and B; ^$^p<0.05 for comparing A and B.

**Table 5:** Multivariable logistic regression analysis of the predicted effect on functional status at the 6 and 12-month follow-up in groups divided on the basis of cognitive problems.

	ADL decline								IADL decline							
	at 6 months				at 12 months				at 6 months				at 12 months			
	Unadjusted		Adjusted		Unadjusted		Adjusted		Unadjusted		Adjusted		Unadjusted		Adjusted	
Independent variables	RR	95% CI	RR	95% CI	RR	95% CI	RR	95% CI	RR	95% CI	RR	95% CI	RR	95% CI	RR	95% CI
Cognitive impairment only (B) vs non-cognitive problem (A)	1.44	0.62–3.36	1.50	0.52–4.39	1.04	0.43–2.50	0.99	0.32–3.06	1.54	0.73–3.20	1.95	0.79–4.78	1.5	0.71–3.14	1.88	0.76–4.66
Delirium superimposed on cognitive impairment (C) vs non-cognitive problem (A)	6.75^&^	1.84–24.78	12.80*	1.65–99.40	6.31^&^	1.89–21.11	7.96*	1.35–46.99	4.13*	1.27–13.46	13.68^&^	1.94–96.55	5.98^&^	1.79–20.03	30.61^&^	2.94–318.54

^*^p<0.05, ^&^p<0.01.

Covariates after adjusting for ADL: age, gender, admission type, type of surgery, hearing impairment, polypharmacy, GDS-15 scores, BMI, risk of malnutrition, CCI scores, pain VAS scores, ASA physical status, length of hospital stay, baseline IADL scores.

Covariates after adjusting for IADL: age, gender, admission type, type of surgery, hearing impairment, polypharmacy, GDS-15 scores, BMI, risk of malnutrition, CCI scores, pain VAS scores, ASA physical status, length of hospital stay, baseline ADL scores.

## Discussion

This prospective study investigated the effects of postoperative delirium on functional status (defined as decline in ADL and IADL scores) in patients who underwent orthopaedic surgery, and examined the interrelationship between underlying cognitive impairment and post-operative delirium. Postoperative delirium was an important predictor for poor IADL but not for poor ADL results. Moreover, delirium superimposed on cognitive problems was a stronger predictor of poor post-discharge functional status than cognitive impairment alone. The risk factors for the development of postoperative delirium identified in this study were in agreement with those identified by previous studies [6]–[10]. However, the incidence of postoperative delirium was lower in the present study than in previous studies, and the difference might be attributed to the higher percentage of elective surgery patients in our study (elective vs emergency surgery: 87.9% vs 12.1%). Moreover, patients in critical condition and unable to give their informed consent, such as some demented patients, were excluded from our study, thereby reducing the incidence of postoperative delirium.

Postoperative delirium had a strong adverse impact on IADL score after controlling for common geriatric problems, comorbidity, polypharmacy, and surgery-related conditions. A previous study reported that delirium had no significant effect on ADL score 6 months after surgery and attributed the relationship between delirium and mortality to some significant underlying medical problems [36]. An association between postoperative delirium and change in functional status at the time of hospital discharge and/or during follow up has also been shown in past studies [37]–[39]. However, the focus of most of these studies is short-term functional status. Although Vida et al. reported that delirium had an adverse effect on long-term IADL functional status 18 months after hospitalization, the effect disappeared after controlling for confounders [40]. In contrast to our results showing a relationship of postoperative delirium to IADL functional decline, those of Edelstein et al. demonstrated that decline in basic ADL but not IADL at 1-year follow-up was more likely in hip fracture surgery patients with postoperative delirium [41]. All the patients in the study by Edelstein et al. underwent hip fracture surgery, which has a greater impact on postoperative delirium and functional status than elective orthopaedic surgery. Furthermore, our study placed more emphasis on geriatric problems as confounders, e.g., malnutrition, polypharmacy, mood status, and symptoms of pain, which impact the risk of postoperative delirium as well as postoperative function. To the best of our knowledge, this is the first study to demonstrate the adverse impact of postoperative delirium on post-discharge ADL and IADL functional status after controlling for most geriatric problems considered as potential confounders. The adverse impact of postoperative delirium in this study was more profound on the IADL than ADL score. The baseline ADL scores for patients developing postoperative delirium and not developing postoperative delirium (85.9±4.81 and 95.6±0.80, respectively) indicated only mild impairment. It implied that the adverse effect of postoperative delirium might be reduced in robust elderly patients. Moreover, the predictive effect of delirium on IADL functional status was only noted 6 and 12 months after surgery. Therefore, factors other than delirium might have a greater effect on short-term functional status of elderly patients undergoing orthopaedic surgery.

Although delirium alone is not predictive for ADL decline during follow-up (after adjusting for confounders such as MMSE), delirium superimposed on cognitive impairment was significantly associated with ADL and IADL decline after surgery. In addition, postoperative delirium was more likely to develop in patients with severe cognitive impairment than in patients with moderate cognitive impairment (mean MMSE scores: group A 26.7±1.8 vs group B 19.8±3.8 vs group C 16.0±4.2). Underlying cognitive impairment (MMSE score <24) alone was not associated with postoperative ADL and IADL decline. The effects of severe cognitive impairment and postoperative delirium were highly interactive. Recovery from postoperative functional impairment by elderly patients undergoing orthopaedic surgery was dependent on the cognitive ability to adhere to rehabilitation programs. Givens et al. reported that each additional cognitive or mood disorder was associated with a greater risk of poor functional status [42]. Therefore, the results of this current study not only confirm the need to identify patients at risk for postoperative delirium, but also the highly interactive nature of the effects of baseline cognitive impairment and superimposed delirium on both ADL and IADL decline.

Despite the effort put into it, this study has several limitations. First, 21.6% (50/232) of the study subjects did not complete follow-up interviews. It has been reported that patients who experience delirium are more likely to miss follow-up appointments [43]. Although the incidence of delirium and the demographic data were similar between patients who completed follow-up and those who did not, there may still be a high risk of systematic attrition bias. Followed-up patients may have poorer clinical outcomes, therefore, the impact of delirium on long-term functional status may be underestimated in these patients. Second, the incidence of postoperative delirium was lower in our study than in previously published studies. Though delirium in this study was detected by the CAM and confirmed by a senior psychiatrist, who avoided overestimation, underestimation was still possible because we excluded patients who were admitted or transferred to ICU or unable to give informed consent, e.g., demented patients. Third, although the baseline cognitive function (assessed on admission before orthopaedic surgery) may be influenced by fracture or pain and lead to overestimation of cognitive impairment, it was not a predictor of ADL and IADL decline in our studies. Fourth, use of a wide range of procedures (performed within a single anatomic region) will impact both the risk of delirium as well as postoperative functional status. Because there were few patients undergoing operations, such as spinal decompression only, other elective knee surgery, and revision hip surgery, we treated similar types of surgery as single covariates, grouping them into four categories (elective spine surgery, elective knee surgery, elective hip arthroplasty, and ORIF/arthroplasty for hip fracture). Further work is needed to investigate the link between postoperative delirium and functional status in patients undergoing orthopaedic surgery at single operative site.

In conclusion, this prospective study evaluated the impact of different cognitive factors on ADL and IADL decline in elderly people undergoing orthopaedic surgery, and it was noted that delirium superimposed on cognitive impairment strongly affects both long-term ADL and IADL functional status. Early identification of patients with baseline cognitive impairment and implementation of strategies to prevent delirium may reduce the likelihood of functional decline following orthopaedic surgery in older patients. Further study is needed to confirm this.
